# Spatial variation and determinant of home delivery in Ethiopia: Spatial and mixed effect multilevel analysis based on the Ethiopian mini demographic and health survey 2019

**DOI:** 10.1371/journal.pone.0264824

**Published:** 2022-03-11

**Authors:** Samuel Hailegebreal, Girma Gilano, Atsedu Endale Simegn, Binyam Tariku Seboka

**Affiliations:** 1 Department of Health Informatics, College of Medicine and Health Science, School of Public Health, Arba Minch University, Arba Minch, Ethiopia; 2 Department of Anesthesia, Wachemo University, Hosaena, Ethiopia; 3 Department of Health Informatics, College of Medicine and Health Sciences, Dilla University, Dilla, Ethiopia; University of Washington, UNITED STATES

## Abstract

**Background:**

Health facility delivery is vital in reducing maternal mortality however, the percentage of deliveries taking place in health facilities were remained below 50%. Therefore, this study was aimed to assess spatial variation and determinant factors of home delivery in Ethiopia.

**Methods:**

We used cross-sectional survey data from Ethiopian Mini Demographics and health 2019. A total of 5,527 reproductive-age women were included in this study. ArcGIS 10.7 was applied to explore the spatial distribution of home delivery and Sat scan 9.6.1 version software was used for spatial cluster analysis. A mixed effect multilevel binary logistic regression model was fitted for determinant factors due to the hierarchical nature of the data. Finally, an adjusted odds ratio (AOR) with 95% confidence level was used to declare significant determinants of home delivery.

**Result:**

According to EMDHS 2019, the spatial variation of home delivery was non-random across the country. Somali, Afar, SNNPR, and part of Amhara were hot spot areas, where some parts of Benishangul, central Oromia, Addis Ababa, Dire Dawa, and Harari were identified as cold spot areas. The odds of women who had primary, secondary and higher education was decreased by 50% (AOR = 0.50; 95% CI: 0.42–0.61), 72% (AOR = 0.28; 95% CI: 0.19–0.40) and 90% (AOR = 0.10; 95% CI: 0.05–0.19) as compared to women no-education respectively. Mothers who had ANC visits were 70% (AOR = 0.30; 95% CI: 0.26–0.36) less likely to have a home birth as compared to women who had no ANC visit. The odds of having home birth among rural residents were 5.2 times (AOR = 5.2; 95% CI: 3.11–8.55) more likely higher compared to the counterpart.

**Conclusion:**

The prevalence of home delivery in Ethiopia was still more than half percent. The spatial distribution was varied across the region. Maternal age, religion, wealth status, had ANC visit, birth order, region, and residence were significant factors with home delivery. Therefore, improving maternal educational status, interventional design in hotspot region, and inspire the mother to take antenatal care is essential to reduce the prevalence of home delivery.

## Introduction

Every year millions of births happen at home without skilled health professional assistance. According to the evidence, only 16% of deliveries were assisted by skilled professionals, while the vast majority (78%) were attended by traditional birth attendants [1**]**. There were many reasons why women prefer home than institution for giving birth. In one study 73.6% of women gave birth at home and there were many factors responsible like lack of a written birth plan for birth alertness and eagerness, an incomplete number of ANC visits and husband preference [2**]**. A recent systematic review showed that home delivery was 48.53% and rural residence, absence of ANC, educational status, age of mothers, and other socio-demographic variables were associated [3–5**]**. The absence of previous Complications, absence of transport access, fear of the surgical procedure, urgent labor, and no difference in giving birth in both paces were other reason [6**]**.

The prevalence of home delivery in the country which varies across the regions. Afar and Somali had a higher and Addis Ababa a lower prevalence of home delivery [7**]**. In another study, home delivery was 36.64% and associated with illiteracy, multi-gravida, history of antenatal care, husband educational status, not owning a radio or television, not pursuing ANC, poor knowledge of obstetric complications, and walking time greater than two hours to health center were the predictors in other studies [8–10**]**. The study conducted in Benishangul Gumuz indicated home delivery was 80% [11]. Previous literature revealed that husband choice place of delivery, mothers’ occupation, late ANC starters, decision making, traditional remedies, and poor knowledge of services were the identified factors [10, 11**]**. Home delivery was further affected positively by ANC, information on birth preparation plan, pregnancies wanted later, not having health insurance, Muslim, and protestant religion; while good wealth index, educational status influenced it negatively [12**]**.

Although the country had a plan of increasing institutional delivery from 20% in 2011 to 60% in 2015, there were a lot of worrying prevalence regarding high home deliveries in the country [13]. Among those prevalence, home delivery mother was 19% in Shashamene in 2016 [14**]**, 44.4% in Achefer district in Amhara region in 2015 [15**]**, 67.6% and 49.3% in Zala district and Anlemo in 2017 [15, 16**]**, and 73.44% from EDHS 2016 [17**]**. And one study identified that participants argue that delivery is a normal life situation and shouldn’t be linked with health problems. Home is a well-thought-out a natural place for delivery and utmost women desire to deliver at home not at health facilities [4]. Overall, the evidence showed that the country’s target is not satisfied and needs more investigations for policymakers and program managers. Therefore, this study aimed to assess spatial distribution and associated factors of home delivery evidence from Ethiopian Mini demographics and health surveys in 2019.

## Methods

### Study design, setting, and period

We used secondary data from Ethiopia Mini Demographic Health Survey (EMDHS) 2019. The EMDHS was a nationally representative study conducted in between EDHS. Ethiopia is located in the Horn of Africa. It has nine regions and two city administrations. Contextually, these regions are categorized as pastoralist region (Benishangul, Somali, Gambella, and Afar), Semi-pastoralist (Oromia, SNNPR), Agrarian (Amhara and Tigray) and City administration (Addis Ababa, Dire Dawa, and Harari). In 2019 EMDHS, the Sample was stratified and selected in two stages. Each region was stratified into urban and rural areas, yielding 21 sampling strata. A total of 9,150 households were selected for the sample, of which 8,794 were occupied. Of the occupied households, 8,663 were successfully interviewed, yielding a response rate of 99%. We salvaged the data from the DHS website (www.dhsprogram.com) after we allowed it by the measure program. A total of 5,527 weighted samples of reproductive-age women who gave birth were included in this study. The detailed sampling procedure was presented in the 2019 EMDHS report [18].

### Study variables

The outcome variable for this study was the place of delivery, which was coded as "0" if the women gave birth at the institution (hospital, health center, or health post) and “1” if the women gave birth at home (birth took place at home).

#### Individual-level (covariates) variable

Maternal education, maternal age, religion, sex of household head, marital status, wealth index, ANC visit, birth order, and parity.

#### Community-level variable

Region, and place of residence.

### Data management and analysis

Statistics were presented as descriptive (frequencies, mean, standard deviations, percentage, or proportions), and spatial and associated factors were examined using (Stata 15, ArcGIS 10.7, and Sat scan 9.6.1) software. Before any statistical analysis, the data were weighted using sampling weight, primary sampling unit, and strata to restore the survey’s representativeness and to account for the sampling design in order to obtain reliable statistical estimates.

### Spatial analysis

We used ArcGIS 10.7 for spatial analyses to explore whether the pattern of home delivery was clustered, dispersed, or randomly distributed in Ethiopia.

### Spatial autocorrelation analysis

The global spatial autocorrelation (Global Moran’s I) was done to assess whether home delivery was dispersed, clustered, or random in the study area. Moran’s, I output ranges between (-1 to +1). Values nearing to −1 designated home delivery were dispersed and that near to +1 designated clustering. A statistically significant Moran’s I (P<0.05) indicate spatial clustering of home delivery.

### Hot spot analysis (Getis-Ord Gi* statistic)

We applied Gettis-OrdGi* statistics to determine in what way spatial autocorrelation of home delivery changes in different locations in the country. Hotspot isolated the statistical significance of the clustering of the event under study-specific area. GI* specified a "hotspot" (high home delivery) and low GI* indicated "cold spot" (low home delivery) [19].

### Spatial interpolation

Spatial interpolation was done to predict home delivery on unsampled areas based on sampled measurement. Various deterministic and geostatistical interpolation methods are also available. Ordinary Kriging and empirical Bayesian Kriging are regarded as the best methods because they incorporate spatial autocorrelation and statistically optimize the weight. Because it had a low mean square error and residual when compared to other interpolation techniques, the ordinary Kriging spatial interpolation method was used for this study to predict home delivery in unobserved areas of Ethiopia.

### Spatial scan statistics

Spatial scan statistics were used to determine statistically significant clusters for home delivery using Kuldorff’s SaTScan version 9.6.1 software. We employed the Bernoulli-based model where home delivery was taken as a case and institutional delivery as a control to fit the model. The default maximum spatial cluster size of < 50% of the population was used. Most likely clusters(primary) and secondary clusters were identified using *p*-values and likelihood ratio tests based on 999 Monte Carlo replications.

### Multilevel analysis

Because the EDHS data is hierarchical, women have been nested within a cluster, and we assume that women in the same cluster may share similar characteristics with women in another cluster. These contradict the logistic regression model’s standard hypothesis of observation independence and equal variance between clusters. This implies that an advanced model must be used to account for cluster heterogeneity. As a result, to investigate factors related to home delivery, a two-level multilevel binary logistic regression model was used. The fitted model was estimated, using xtmelogit function as shown:

$$\text{log}\;\left(\frac{\pi_{\text{ij}}}{1-\pi_{\text{ij}}}\right)=\beta_{0}+\beta_{1}{\text{X}}_{\text{ij}}+\beta_{2}{\text{Z}}_{\text{ij}}+uij;$$

where, i and j are the level 1 (individual) and level 2 (community) units, correspondingly; X and Z refer to individual and community-level variables, in sequence. πij is the probability of the home delivery for the i^th^ mother in the j^th^ community. To check the nested nature of the data, Intra-class Correlation Coefficient (ICC) quantifies the degree of heterogeneity of home delivery between clusters (the proportion of the total observed variation in home delivery that is attributable to between cluster variations) as $\text{ICC}=\frac{{\sigma^{2}}_{a}}{{\sigma^{2}}_{a}+{\sigma^{2}}_{b}}$; where σ^2^_*a*_ is the community level variance and σ^2^_*b*_ indicates individual-level variance. The individual variance (σ^2^_*b*_) is equal to π^2^/3 that is the fixed value. Likelihood Ratio (LR) test for model comparison and deviance (-2LL) for the goodness of fit were calculated, while Median Odds Ratio (MOR) and Proportional Change in Variance (PCV) were also estimated [20].

Four models were built in sequence; First, we built a model with only outcome variable to acquire nature of variation (null model); second, we built a model with individual-level factors (model-I); third was a model with community-level (model-II) factors; finally, a mixed-effect model with both individual and community level (model-III) factors was fitted. To include variable in the model, p-value < 0.25 and to declare association p-value<0.05 were used. AOR with 95% CI was also used to articulate the results.

### Ethical consideration

Since we used secondary publicly available survey data from the MEASURE DHS program, ethical approval and participant consent were not necessary for this particular study. We requested DHS Program and permission was granted to download and use the data for this study from http://www.dhsprogram.com. The EMDHS data collection obtained permission from Ethiopian Health Nutrition and Research Institute (EHNRI) Review Board and the National Research Ethics Review Committee (NRERC) at the Ministry of Science and Technology.

## Results

### Socio-demographic characteristics of study participant

A total of 5,527 reproductive-age women who gave birth were included in this study. The majority of the participants were from Oromia 2211 (40%) and the minority participant 16 (0.30%) were from Harari region. Regarding religion, 2100 (38%) of the respondents were Muslim and 1860 (33.7%) were orthodox Christian followers. More than half, of the study participants, were in the age group of 15–29 3050(55.19%) years. More than half, (53%) of respondents had no education. The majority of the respondents (75.27%) were rural residents and over 95% were married (Table 1).

**Table 1:** Socio-demographic characteristics of the study population (Weighted, N = 5527).

	Frequency	Percent
**Region**		
Pastoralist region	587	9.50
Agrarian	1421	26.60
Semi-pastoralist	3317	60.00
City administration	202	3.60
**Residence**		
Rural	4,160	75.27
Urban	1,367	24.73
**Age**		
15–29	3050	55.19
>=30	2476	44.81
**Sex household head**		
Male	4776	86.42
Female	751	13.58
**Religion**		
Orthodox	1860	33.70
Muslim	2100	38.0
Protestant and others	1566	28.30
**Marital status**		
Single	234	4.24
Married	5293	95.76
**Educational status**		
No-education	2962	53.58
Primary	1956	35.40
Secondary and Higher	609	11.02
**Wealth status**		
Poorest	1321	23.89
Poorer	1198	21.67
Middle	1044	18.88
Richer	960	17.36
Richest	1005	18.19
**Birth order**		
1^st^	1204	21.79
2–3	1765	31.93
4–5	1237	22.39
6 and above	1320	23.89

### Prevalence of home delivery

**Fig 1:**
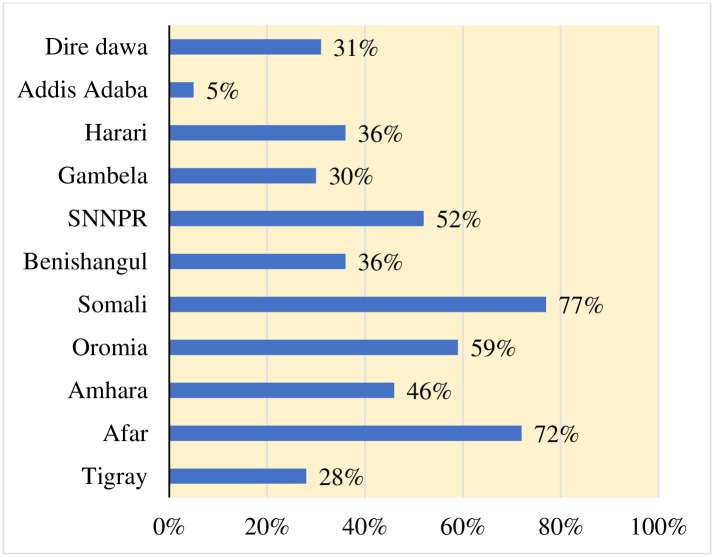
Regional prevalence of home delivery in Ethiopia in 2019.

The prevalence of home delivery was 52.50% [95%, CI: 0.51 0.54] in Ethiopia in 2019. Which showed that significant variation across the region ranging from 5.2% in the Addis Ababa region to 76.7% in Somali (Fig 1). Regarding to place of residence the highest prevalence of home delivery was observed in rural (59.9%) area.

### Spatial analysis of home delivery in Ethiopia

#### Spatial autocorrelation analysis

**Fig 2:**
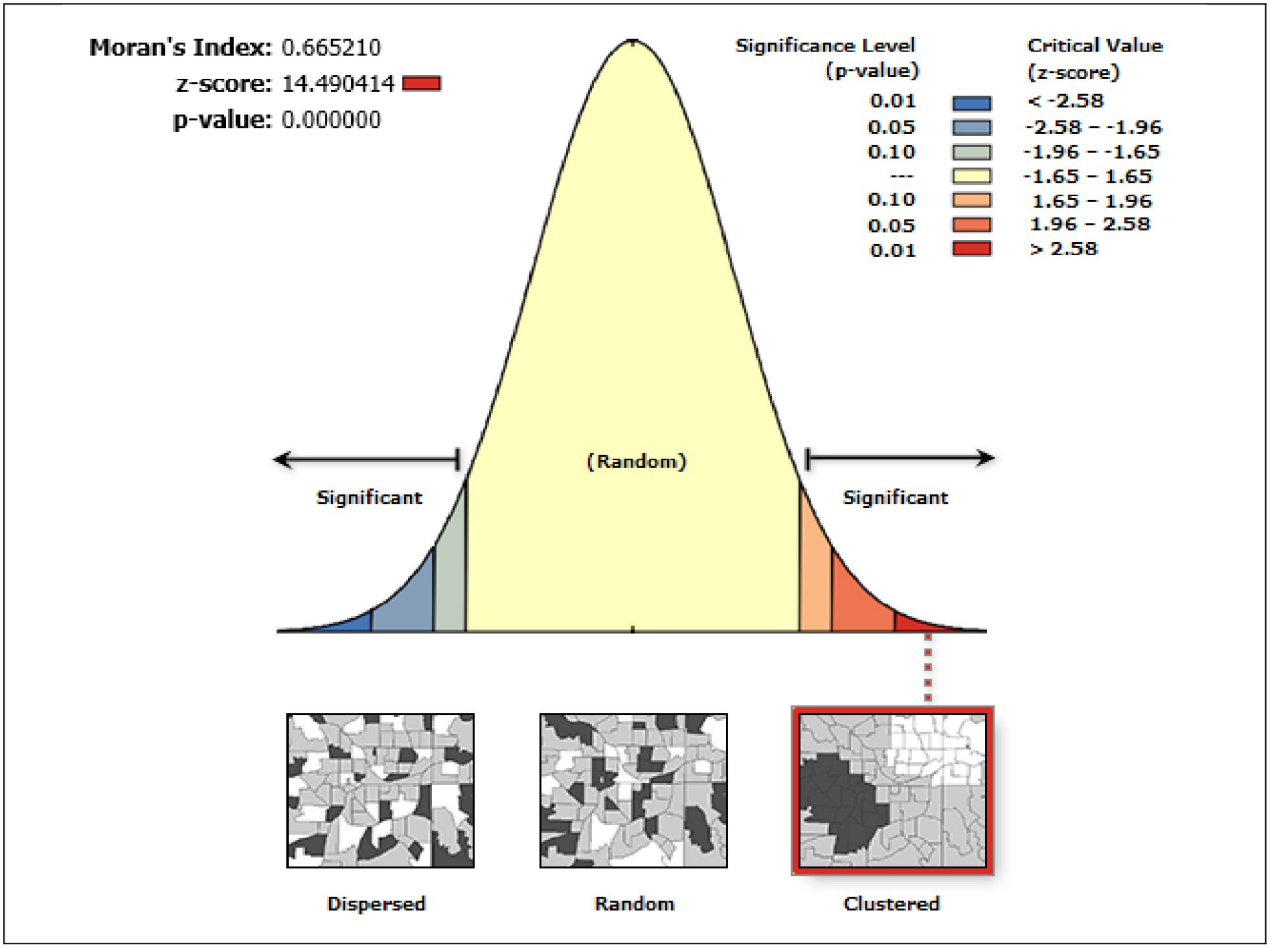
Spatial autocorrelation of home delivery in Ethiopia; 2019.

Spatial variation of home delivery in Ethiopia has significantly varied in EMDHS 2019 analysis across the country. The global Moran’s I value was 0.665 (*p*-value < 0.001) with a Z-score of 14.5(Fig 2).

#### Incremental spatial autocorrelation

**Fig 3:**
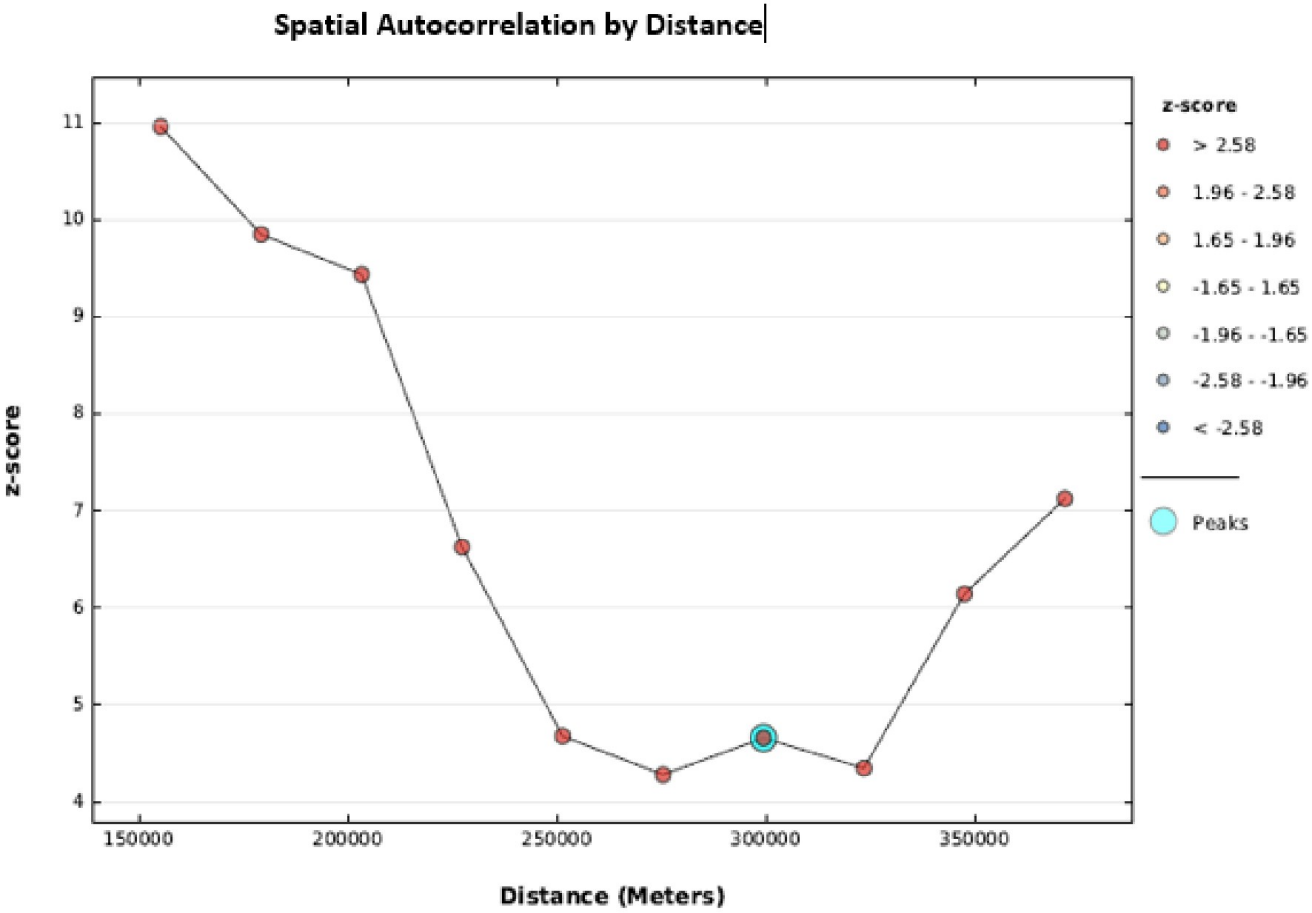
Incremental spatial autocorrelation of home delivery in Ethiopia.

Incremental spatial autocorrelation is a series of line graphs and their corresponding z-scores. The Z-score reflected the strength of clustering, and the peak was statistically significant at 24.04 km indicated where the spatial processes were promoted sound clustering. A total of 10 distance bands were detected with the beginning distance of 155,190 meters (Fig 3).

#### Hotspot analysis of the survey

**Fig 4:**
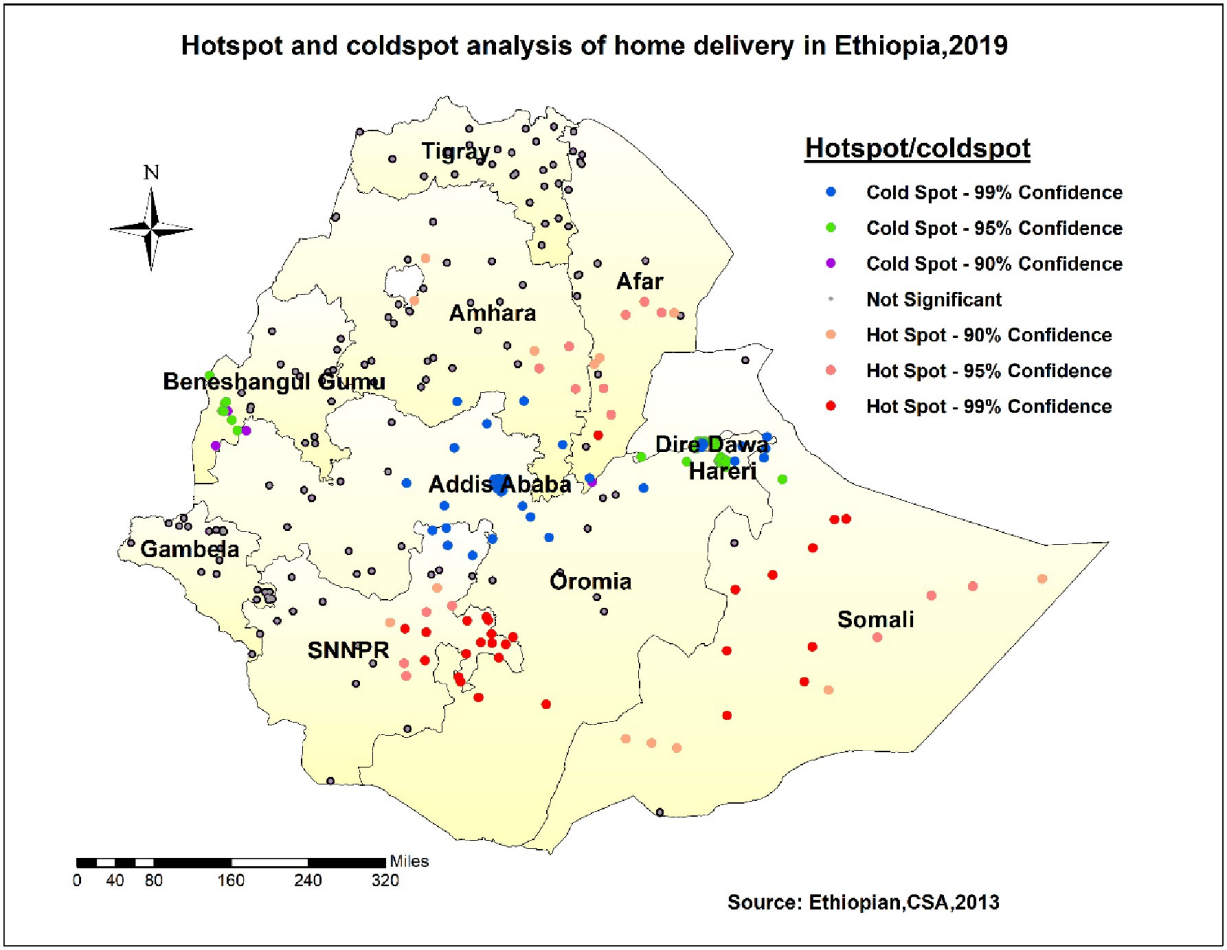
Hotspot/Cold spot analysis of home delivery in Ethiopia, 2019.

In EMDHS-2019 sampled data, Somali, Afar, SNNPR, and parts of Amhara were identified as hot spot (high risk) regions for home delivery. In contrast, some part of Benishangul, central Oromia, Addis Ababa, Dire Dawa, and Harari were identified as cold spot (low risk) regions (Fig 4).

#### Spatial interpolation

**Fig 5:**
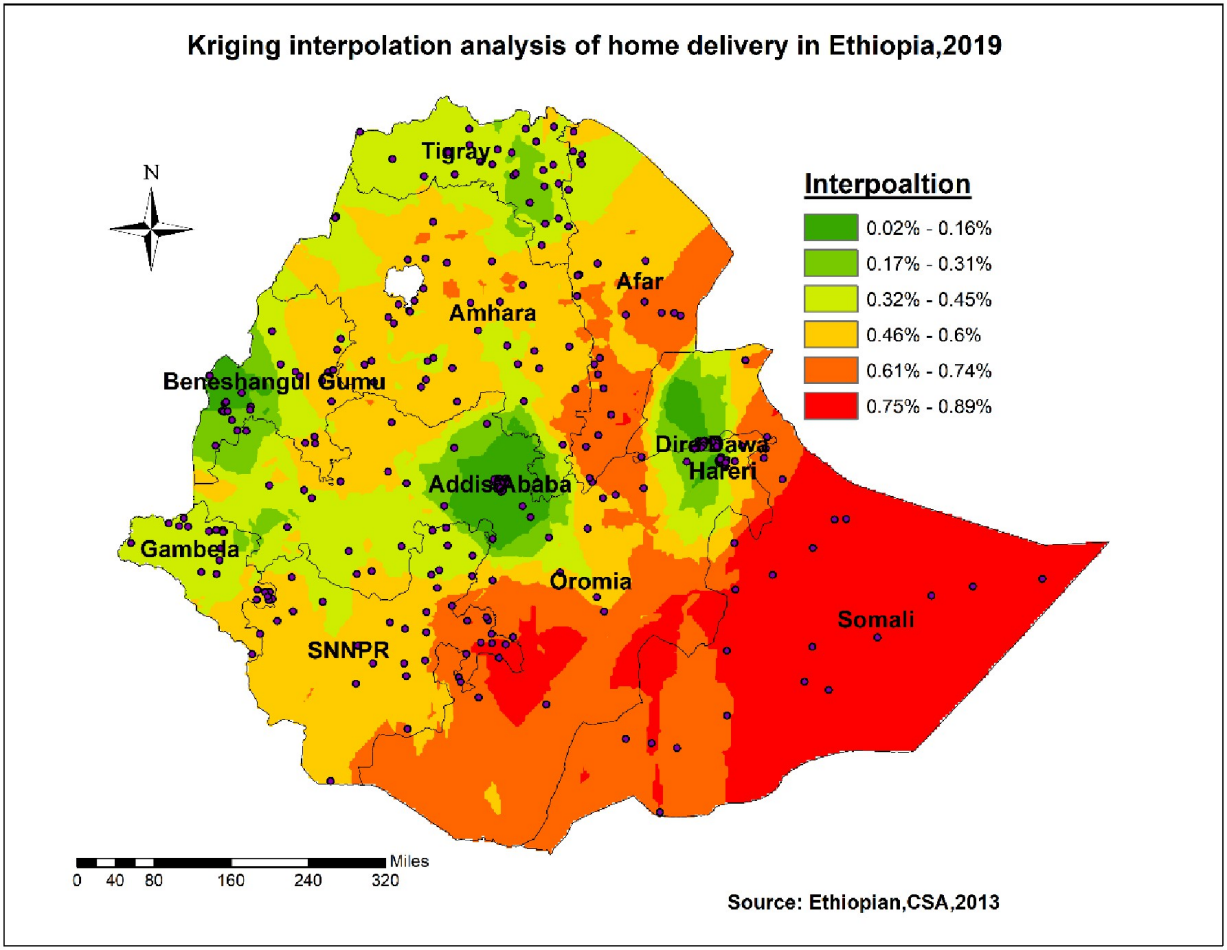
Ordinary kriging spatial interpolation of home delivery in Ethiopia, 2019.

The ordinary Kriging spatial interpolation revealed; that some borders of SNNPR, Afar, southern Oromia, and Somali regions had shown a high proportion of home delivery. In contrast, a low proportion of home delivery was identified in central Oromia, Benishangul, Dire Dawa, Harari, and parts of Tigray regions (Fig 5).

#### Spatial scan statistical analysis

**Fig 6:**
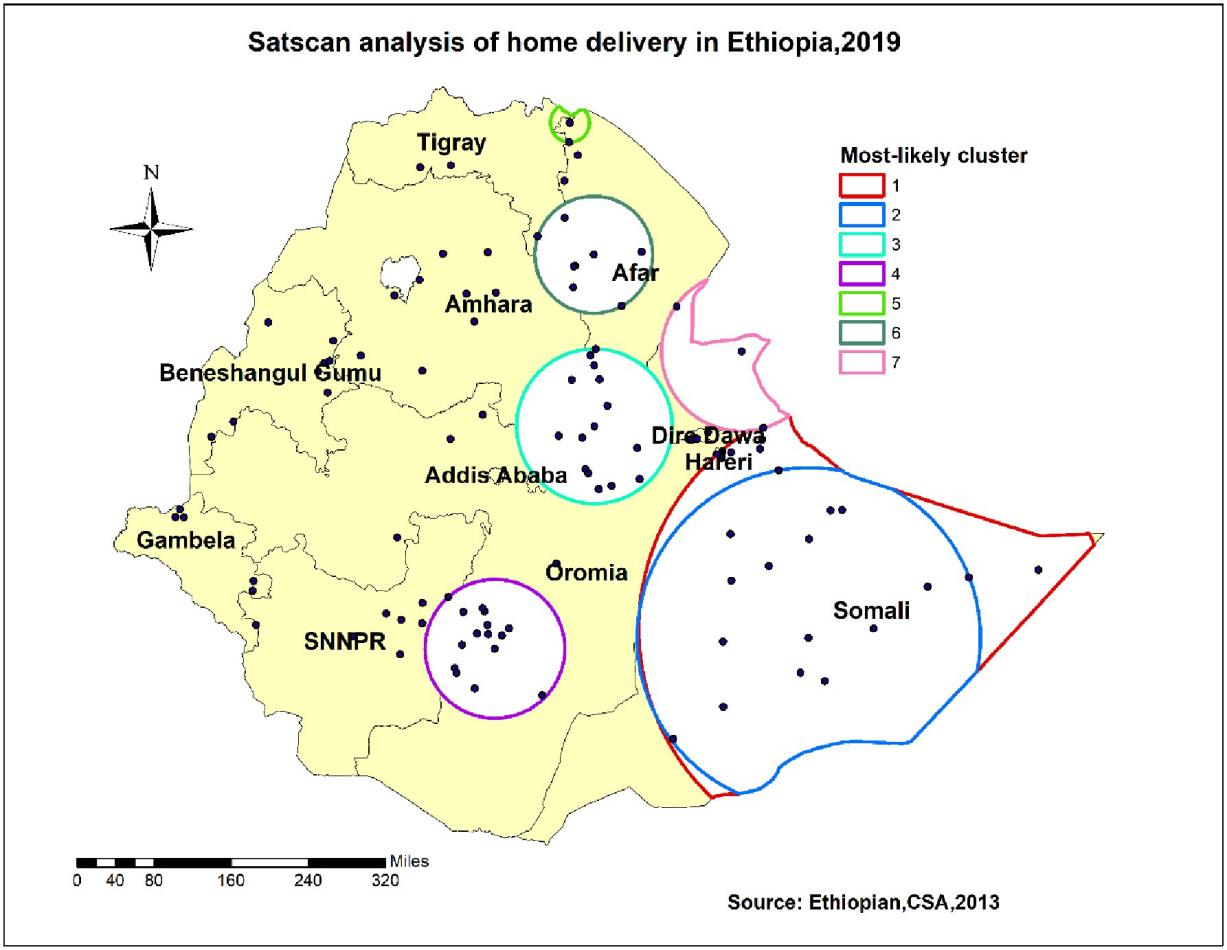
Spatial StatsCan analysis of home delivery in Ethiopia, 2019.

The spatial scan statistics showed a total of 87 significant clusters, of which 27 were most likely/primary clusters and 60 were secondary clusters. The most likely cluster was located in Harari, border of Oromia and Somali regions which was centered at 6.639662 N, 44.465853 E with a 390.28 km radius. The log-likelihood ratio: 187.827676; and Relative risk was 1.80 with P-value <0.01(Fig 6). It means, that women in the spatial circle had 1.8 times the risk of home delivery than women outside the spatial circle.

#### Random effect analysis

The community-level variance [community-level variance = 5.56, Standard Error (SE) = 0.65, P-value <0.001], showing the existence of significant difference between clusters concerning home delivery. This difference was supported by ICC of 62% in the null that indicted the variation of home delivery was attributable between cluster variability. In addition, the final model showed (model3) 76% of the variation of home delivery was attributable to both individual and community-level predictors. Finally, model fitness was assessed by deviance (-2LL); and the model with the lowest deviance value was the best-fitted model (Table 2).

**Table 2:** Random effect analysis result.

Parameter	Null model	Model I	Model II	Model II
Community-level variance (SE)	5.6 (0.65)	1.6 (0.22)	2.2 (0.27)	1.2 (0.18)
Loglikelihood	-2863	-2498.94	-2747.18	-2449
Deviance	5726	4997.88	5494.36	4898
MOR	6.1	3.3	3.8	2.8
PVC (%)	Ref	71.4	61	76
ICC	0.63	0.33	0.40	0.28

#### Determinant of home delivery

Both individual and community-level factors were presented simultaneously in (Table 3). Maternal age, religion, wealth status, had ANC visit, and birth order from individual-level factors, and region, and place of residence were significantly associated with home delivery.

**Table 3:** Factors associated with home delivery in Ethiopia, EMDHS 2019.

Variables	Model 0	Model 1	Model 2	Model 3
**Respondent age**				
15–29	**-**	Ref	**-**	Ref
30–39	**-**	0.73[0.59–0.90] **	**-**	0.77[0.63–0.95] *
40–49		0.77[0.55–1.09]	**-**	0.86[0.61–1.22]
**Religion**				
Orthodox	**-**	Ref	**-**	Ref
Muslim	**-**	1.2[0.85–1.61]	**-**	0.74[0.50–1.09]
Protestant	**-**	1.7[1.20–2.35] **	**-**	1.50[1.04–2.16] *
Others		2.1[1.11–3.86] *		1.84[0.98–3.46]
**Marital status**				
Single	**-**	Ref	**-**	Ref
Married	**-**	0.66[0.23–1.92]	**-**	0.66[0.23–1.92]
Widowed	**-**	0.58[0.16–2.08]	**-**	0.59[0.17–2.13]
Divorced	**-**	0.74[0.24–2.31]	**-**	0.81[0.26–2.53]
**Educational status**				
No-education	**-**	Ref	**-**	Ref
primary	**-**	0.49[0.40–0.59] **	**-**	0.50[0.42–0.61] **
Secondary	**-**	0.26[0.18–0.38] **	**-**	0.28[0.19–0.40] **
Higher		0.09[0.04–0.38] **	**-**	0.10[0.05–0.19] **
**Sex of household head**				
Male				Ref
Female		0.98[0.78–1.23]		0.90[0.73–1.16]
**Wealth status**				
Poorest	**-**	Ref	**-**	Ref
Poorer	**-**	0.59[0.46–0.75] **	**-**	0.66[0.52–0.84] **
Middle		0.57[0.44–0.74] **		0.64[0.49–0.84] **
Richer	**-**	0.28[0.21–0.38] **	**-**	0.35[0.26–0.47] **
Richest		0.11[0.08–0.16] **		0.23[0.16–0.35] **
**Had ANC visit**				
No				Ref
Yes		0.29[0.25–0.34] **		0.30[0.26–0.36] **
**Birth order**				
1^st^	**-**	Ref	**-**	Ref
2–3	**-**	1.74[1.33–2.26] **	**-**	1.70[1.32–2.26] **
4–5	**-**	2.12[1.47–3.06] **	**-**	2.00 [1.41–2.94] **
6+	**-**	2.33[1.32–4.11] **	**-**	2.30[1.28–4.02] **
**Parity**				
≤ 2	**-**	Ref	**-**	Ref
3–5	**-**	1.13[0.86–1.48]	**-**	1.13[0.86–1.49]
≥ 6+	**-**	0.94[0.56–1.57]	**-**	0.91[0.54–1.52]
Community-level variables				
**Region**				
		-		
Tigray		-	Ref	Ref
Afar		-	18 [6.98–45] **	9.00[3.68–20.56] **
Amhara		-	2 [1.02–5.85] *	2.00 [1.03–4.36] *
Oromia		-	5 [1.95–10.9] **	3.50 [1.65–7.47] **
Somali		-	39 [14.86–99] **	10 [4.24–24.69] **
Benishangul		-	1 [0.47–3.09]	0.94 [0.42–2.10]
SNNPR		-	3 [1.48–8.32] **	2.00 [0.99–4.49]
Gambella		-	3 [1.07–6.94] *	2.20 [0.97–4.92]
Harari		-	2 [0.77–5.72]	2.90 [1.18–7.11] *
Addis Ababa			1 [0.36–4.00]	2.00 [0.72–6.09]
Dire dawa		-	2 [0.73–5.68]	2.20 [0.86–5.52]
**Residence**			18 [0.73–5.68]	
Urban		-	Ref	Ref
Rural		-	18 [10.4–31.55] **	5.20 [3.11–8.55] **

*Key: Ref: Reference group; p-value 0.05–0.01 *: p-value < 0.01 **.

The odds of having home delivery among women of age group 30–39 were decreased by 23% (AOR = 0.77; 95% CI: 0.63–0.95) compared to 15–29. The odds of home delivery among protestant religion followers had 1.5 (AOR = 1.5; 95% CI: 1.04–2.16) times higher compared to orthodox religion follower. The odds of home delivery among women who had primary, secondary and higher education were decreased by 50% (AOR = 0.50; 95% CI: 0.42–0.61), 72% (AOR = 0.28; 95% CI: 0.19–0.40), and 90% (AOR = 0.10; 95% CI: 0.05–0.19) compared to women with no-education respectively.

The odds of home delivery among women with poorer, middle, richer, and richest wealth status were 34% (AOR = 0.66; 95% CI: 0.52–0.84), 32% (AOR = 0.64; 95% CI: 0.49–0.84]), 65% (AOR = 0.35; 95% CI: 0.26–0.47), and 77% (AOR = 0.23; 95% CI: 0.16–0.35) less likely to give birth at home as compared to poorest wealth status women. Mothers who had ANC visit were 70% (AOR = 0.30; 95% CI: 0.26–0.36) less likely having home birth as compared women who had no ANC visit.

The odds of home delivery among the women who had birth order of 2–3 were 1.7 times (AOR = 1.7; 95% CI: 1.32–2.26), 4–5 were 2 times (AOR = 2; 95% CI: 1.41–2.94), and 6and above were 2.3 times (AOR = 2.3; 95% CI: 1.28–4.02) more likely to deliver home compared to women who had one birth order.

Regarding region, women from Somali (AOR = 10; 95% CI: 4.24–24.69), Afar (AOR = 9; 95% CI: 3.68–20.56), Oromia (AOR = 3.5; 95% CI: 1.65–7.47), Harari (AOR = 2; 95% CI: 1.41–2.94), and Amhara (AOR = 2; 95% CI: 1.03–4.36) had higher odds of home delivery compared to Tigray region. The odds of home births among rural residents were 5.2 times (AOR = 5.2; 95% CI: 3.11–8.55) more likely higher compared to the counterpart.

## Discussion

This study was aimed to explore the spatial distribution and associated factors of home deliveries among reproductive-age women in Ethiopia. The study showed that 52.5% (95%, CI: 0.51, 0.54) of women gave birth at home in 2019. The finding showed home delivery was decreased as per the evidence previous 5, 2011 and 2016 EDHS in Ethiopia [21]. This finding was higher than that of the study conducted in Sub-Sharan African which was 44% [22]. It was lower than the finding from a study conducted in Afar, Ethiopia that was 71% [23]. The finding was also higher than that of a study done in Shashemene [24]. This discrepancy might be due to the study setting, time variation; and sample size difference where women’s attitudes, cultural differences could be the possible reasons.

The geospatial distribution of home delivery was non-random in Ethiopia in 2019. Somali, Afar, SNNPR, and part of Amhara were identified as hot spot regions, whereas some parts of Benishangul, central Oromia, Addis Ababa, Dire Dawa, and Harari were identified as cold spot regions for home delivery. Besides, spatial sat scan analysis revealed that the most likely/primary cluster was located in Harari, borders of Oromia, and Somali regions. The variation might be due to cultural influence and infrastructure limitations [17]. Also, it might be that regions such as Addis Ababa, central Oromia, Dire Dawa, and Harari were urban areas where most mothers had better access to the media and healthcare facilities than other regions. For Benishangul region, different activities that encourage women to deliver at health facilities may help them deliver at institutions. This could be because the Ethiopian Federal Ministry of Health and other stakeholders, such as UNICEF, placed a high priority on the issue. Furthermore, the fact that the region had to host a large number of refugees aided in attracting more attention [25].

In multilevel analysis maternal age, religion, educational status, wealth status, had ANC visits, and birth order from the individual level(level1) and region and residence from community level(level2) factor were significantly associated with home delivery. From individual-level factors women who didn’t attain formal education had higher likelihood of home delivery than women who had formal education, the finding is consistent with previous studies [17, 21, 26, 27]. This might be due to the optimistic attitude of educated women and that might be enhanced women’s self-determination; the knowledge of the drawback of home delivery. The odds of home birth among women in the age group of 30–39 were lower than that of 15–29. This finding was similar to previous studies done in Ethiopia [1] Zambia [28], and Tanzania [27]. The reason might be due to the young women’s fear of complications during home delivery, and it might be also due to ignorance of older women.

This study showed that the poorest wealth status increased the likelihood of home delivery. This is consistent with similar, previous studies [29, 30]. This might be reasoned that the economic capability of the households and costs related to transport might influence the preference of place of delivery. Besides, these mothers from higher wealth status might be pursuing maternal health service than others. The number of ANC visits was also associated with home birth; women who had ANC visits less likely to give birth at home. This is consistent with previous studies [17, 31]. The reason might be the exposure of women to health education related to home delivery given by health care professionals. The odds of home delivery was increased with higher birth order., This is consistent with other studies [17, 28]. This might be due to the poor quality of services during the preceding births embarrassing settings and shortage of skilled health care providers, and erratic supplies of drugs and equipment were the possible explanations.

The result of mixed effect multilevel logistic regression analysis of community-level factors revealed that place of residence and region were significant predictors. Women living in rural areas had higher odds of home delivery than their counterparts. the finding is comparable with others studies [32–34]. The possible reasons might be the home deliveries among these cohorts of women might have financial and topographical barriers to accessing institutional/skill personnel assistance. Furthermore, mothers who live in the pastoral region (Somali and Afar), Agrarian region (Harari and Amhara) had higher odds of home delivery compared to Tigray region, and this result is consistent with other studies [17, 33]. The possible explanation might be the inaccessibility of health facilities in pastoral regions and People might have difficulty having permanent residency access to the services.

### Strength and limitation of the study

The current study used a large data set and represented the whole country. In addition, advanced methodologies like mixed effect multilevel and spatial analysis were applied to get the appropriate standard error and risky area of home birth in Ethiopia. However, the cross-sectional nature of the survey, some important factors like, median exposure, and obstetrics factors are not included in the analysis.

## Conclusion

Based on the findings of the survey, it was concluded that the overall prevalence of home delivery was more than half. The spatial distribution was varied across the region. This study identified hot spot area of home delivery in Somali, Afar, SNNPR, and parts of Amhara. Maternal age, religion, wealth status, had ANC visit, birth order, region, and residence were significantly influenced home delivery. These findings contribute to the understanding of the realistic view of home delivery spatial variability in the country and factors to alleviate home delivery. Therefore, improving maternal education, interventional design in hotspot regions, and inspire the mother to take antenatal care might be essential to reduce the prevalence of home delivery in Ethiopia.

## Abbreviations

ANC: Antenatal Care
AOR: Adjusted Odds Ratio
CI: Confidence Interval
CSA: Central Statics Agency
DHS: Demographic and Health Survey
EMDHS: Ethiopia Mini Demographic and Health Survey
ICC: Intra Class Correlation Coefficient
LLR: Log-Likelihood Ratio
MOR: Median odds ratio
OR: Odds Ratio
PVC: Proportional Change in Variance
RR: Relative Risk
SNNPR: Southern Nations, Nationalities, and Peoples’ Region
WHO: World Health Organization
